# *GmWRKY33a* is a hub gene responsive to brassinosteroid signaling that suppresses nodulation in soybean (*Glycine max*)

**DOI:** 10.3389/fpls.2024.1507307

**Published:** 2025-01-16

**Authors:** Mingliang Yang, Chengjun Lei, Chao Ma, Xiuming Hou, Mingming Yao, Liang Mi, Enliang Liu, Linli Xu, Shukun Wang, Chunyan Liu, Qingshan Chen, Dawei Xin, Chang Xu, Jinhui Wang

**Affiliations:** ^1^ Heilongjiang Green Food Science Research Institute, Northeast Agricultural University, Harbin, Heilongjiang, China; ^2^ National Key Laboratory of Smart Farm Technologies and Systems, Key Laboratory of Soybean Biology in Chinese Ministry of Education, College of Agriculture, Northeast Agricultural University, Harbin, Heilongjiang, China; ^3^ Grain Crops Institute, XinJiang Academy of Agricultural Sciences, Urumqi, Xinjiang Uygur, China

**Keywords:** soybean, symbiosis, brassinosteroids (BRs), RNA-Seq, WGCNA, GmWRKY33a

## Abstract

Brassinosteroids (BRs) are key phytohormones influencing soybean development, yet their role in symbiosis remains unclear. Here, the RNA-Seq was used to identify important gene associated with BRs and symbiotic nitrogen fixation, and the function of candidate gene was verified by transgenic hairy roots. The result shows that the RNA-Seq analysis was conducted in which BR signaling was found to suppress nodule formation and many DEGs enriched in immunity-related pathways. WGCNA analyses led to the identification of *GmWRKY33a* as being responsive to BR signaling in the context of symbiosis establishment. Transgenic hairy roots analyses indicated that GmWRKY33a served as a negative regulator of the establishment of symbiosis. The qRT-PCR analysis confirmed that BR signaling upregulates *GmWRKY33a*, leading to nodulation suppression and activation of soybean immune responses. In summary, our research revealed that BR suppresses root nodule formation by modulating the immune signaling pathway in soybean roots. We further identified that GmWRKY33a, a crucial transcription factor in BR signaling, plays a negative role in the symbiotic establishment.

## 1 Introduction

Soybean (*Glycine max* L. Merr.) serves as a major source of vegetable oil and protein for human consumption, in addition to being a cornerstone crop for the development of sustainable agriculture (Ma et al., 2023; Wang et al., 2023a). Current soybean production efforts, however, require large amounts of industrial nitrogen-based fertilizers, thus posing a substantial threat to soil microbes and the overall soil environment (Wang et al., 2023b). Soybean plants can establish a symbiotic relationship with rhizobia that results in the efficient conversion of atmospheric nitrogen into a form that can be used to support growth and development in a mutually beneficial manner (Ma et al., 2024a). As soybean production is forecast to grow by 55% as of 2050 and symbiotic nitrogen fixation can provide large volumes of nitrogen to fuel soybean growth, the key importance of this symbiotic nitrogen fixation capacity will be increasingly relevant to agricultural productivity and sustainability (Ciampitti et al., 2021).

Nodule development necessitates signaling interactions between leguminous plants and the associated rhizobia, together with the orchestration of complex programs of gene regulation within these legumes (Roy et al., 2020). In response to the detection of host-derived flavonoids, rhizobia can secrete a variety of nodulation factors (NFs) (Denarie and Cullimore, 1993; Zhang et al., 2009). Symbiotic receptors including Nod factor receptor 1 (NRF1) and Nod factor receptor 5 (NRF5), in turn, allow the legume hosts to respond to these NFs (Limpens et al., 2003; Madsen et al., 2003; Radutoiu et al., 2003; Smit et al., 2007), which induce a spike in nuclear calcium signaling that is decoded by calmodulin-dependent protein kinase (CCaMK) (Mitra et al., 2004; Gleason et al., 2006), leading to the phosphorylation and activation of CYCLOPS (Yano et al., 2008; Singh et al., 2014; Rudaya et al., 2022). The resultant signals drive the upregulation of a series of symbiotic downstream genes involved in the common symbiosis signaling pathway (CSSP), including *Ethylene Responsive Factor Required for Nodulation 1* (*ERN1)* and *Nodule Inception* (*NIN*) (Cerri et al., 2017; Roy et al., 2020). The transcription factor Nodulation Signaling Pathway 1 (NSP1) is capable of binding the promoter regions upstream of both *ERN1* and *NIN* to activate their expression, with its transcriptional activity being subject to Nodulation Signaling Pathway 2 (NSP2)-mediated regulation in the context of symbiosis (Hirsch et al., 2009; He et al., 2021). To date, the molecular and genetic mechanisms that underlie the establishment of this symbiotic relationship remain poorly understood.

As with all other plant developmental processes, nodulation is regulated by phytohormones (Yang et al., 2011; Vriet et al., 2013; Planas-Riverola et al., 2019). Brassinosteroids (BRs) are polyhydroxylated steroid phytohormones that are detected by the BR Insensitive 1 (BRI1) membrane receptor (Wang et al., 2001; Kinoshita et al., 2005) and BRI1-associated kinase (BAK1) as a co-receptor (Li and Chory, 1997; Vicentini et al., 2009; Sun et al., 2013), allowing for the regulation of a diverse array of plant developmental processes. BR signaling is transmitted from the membrane to the nucleus through complex and dynamic interactions between BRI1 Suppressor 1 (BSU1) (Tang et al., 2008; Wang et al., 2011), BR-insensitive 2 (BIN2) (Kim et al., 2009, 2011; Wang et al., 2012), and Brassinazole-Resistant 1/BRI1-EMS-Suppressor 1 (BES1/BZR1) (Wang et al., 2002; Yin et al., 2002). When BR signaling is initiated, BIN2 is degraded, leading BES1 and its homologs to be dephosphorylated whereupon they accumulate in the nucleus and control the expression of genes related to BR responses (Zhu et al., 2017). In addition to shaping plant growth and development, BR signaling processes are central to the orchestration of microbial stress responses (De Bruyne et al., 2014; Wei and Li, 2016). BRs can enhance the ability of plants to resist a wide range of pathogens. While BR-mediated disease resistance (BDR) is evident following BR treatment (Nakashita et al., 2003), its precise mechanistic basis remains poorly understood.

In different legume species, the precise roles that BRs play in the establishment of symbiotic relationships vary (Chen et al., 2023). In *M. truncatula* and *Pisum sativum*, *Mtbri1* mutants lacking the BR receptor present with fewer nodules (Ferguson et al., 2005; Cheng et al., 2017). The *lk* (5α reductase-deficient) and *lkb* (sterol C-24 reductase-deficient) mutant pea lines exhibiting impaired BR biosynthesis similarly exhibit reductions in nodule numbers (Ferguson et al., 2005). The application of BRs to leaves following rhizobial inoculation in *Pisum sativum* is associated with significantly higher root nodule numbers (Shahid et al., 2011). While these results emphasize the positive roles that BRs can play in nodulation for certain leguminous species, the leaf application of BRs to *Phaseolus vulgaris* or the BR inoculation treatment of *Lens culinaris* were associated with impaired nodule formation (Upreti and Murti, 2004). BR signaling can negatively regulate NF signaling activity in soybean plants, adversely affecting the establishment of symbiosis (Chen et al., 2023). When brassinaz, an inhibitor of BR biosynthesis, was exogenously applied to the Enrei cultivar, this led to better nodule formation, whereas the application of BRs to the roots of these plants effectively suppressed nodulation (Hunter, 2001). Overexpressing *BES1/BZR1* and homologs thereof has similarly been shown to decrease the formation of root nodules in soybean (Yan et al., 2018). The key BR signaling pathway component GmBES1-1 has been shown to inhibit nodulation through its interactions with GmNSP1 and GmNSP2 and the inhibition of the ability of GmNSP1s to bind DNA (Chen et al., 2023). BRs are central regulators of plant immune function, and the effective establishment of rhizobial symbiosis necessitates a homeostatic balance between signals conducive to antimicrobial immunity and symbiosis (Wei and Li, 2016). Beyond their ability to directly affect NF signaling activity during the establishment of symbiosis, BRs may also be capable of regulating the immune response to shape nodulation in this context, although the underlying mechanisms through which BR signaling can modulate immunity in this setting remain unknown.

WRKY zinc-finger motif-containing proteins frequently function in the coordination of defense responses in *Arabidopsis* (Eulgem and Somssich, 2007; Liu et al., 2018), with defense signaling or pathogen infection frequently provoking the upregulation of large numbers of WRKY genes that activate an array of downstream genes linked to disease resistance including *NPR1* and *PRs* (Li et al., 2020). WRKY33 is among the best-studied members of this family, with several studies having documented its mechanistic importance in response to biotic stressors (Zheng et al., 2006; Liu et al., 2017). WRKY33 can also mediate pathogen-associated responses in plants such that, when overexpressed, it can engender greater host resistance against many microbes (Liu et al., 2015). *WRKY33* is capable of responding to pathogen infection through its ability to modulate calcium ion, calmodulin, and hormone signaling (Zhou et al., 2020), in addition to shaping redox homeostasis (Zheng et al., 2006) and autophagy (Lai et al., 2011). MAPK and CDPK-dependent WRKY33 phosphorylation upstream of pathogen infection also shapes its functional effects (Mao et al., 2011; Zhou et al., 2020). Beyond these pathogen-induced responses, WRKY33 also shapes the ability of plants to tolerate abiotic stressors including salinity (Jiang and Deyholos, 2009), hypoxia (Tang et al., 2021), heat (Li et al., 2011), and cold stress (Guo et al., 2022). While there have been several studies analyzing soybean *GmWRKY33* functions in the context of pathogen resistance, its impact on symbiotic signaling has yet to be established.

Here, treatment with eBL (Epibrassinolide) was found to significantly reduce root nodule numbers and to promote the expression of defense-associated genes including PRs. Through subsequent RNA-seq analyses, differentially expressed genes following eBL treatment were found to be enriched in nodulation and plant-pathogen interaction-related pathways. WGCNA and qPCR analyses led to the identification of *Glyma.09G280200*, which encodes a GmWRKY33 protein, as a hub gene involved in coordinating the BR signal transduction processes related to symbiosis establishment. This gene was designated *GmWRKY33a*. Through transgenic analyses, *GmWRKY33a* was found to negatively regulate symbiosis, with BRs suppressing nodulation through the promotion of GmWRKY33a expression. Together, these findings offer new insight into BR signaling processes and their role in symbiosis establishment and efficient nitrogen fixation in soybeans.

## 2 Materials and methods

### 2.1 Materials and cultivation conditions

*Sinorhizobium fredii* HH103 (hereafter HH103) and GUS-tagged HH103 (HH103-GUS) (Wang et al., 2023a) were utilized to conduct all nodulation experiments described herein. Both HH103 and HH103-GUS were cultured in TY medium with appropriate antibiotics (50 μg/mL) at 28°C. Soybean plants were cultivated in a greenhouse (16 h light/8 h dark, 25°C).

### 2.2 Inoculation and BR sensitivity assays

After using Cl_2_ (generated from 96 mL sodium hypochlorite and 4 mL concentrated hydrochloric acid in drying bottle) to sterilize the surfaces of Dongnong 50 (DN50) seeds, they were sown in vermiculite in plastic jars that had been autoclaved. Irrigation was performed with a nitrogen-deficient nutrient solution. HH103 or HH103-GUS were cultured in TY medium to an OD_600_ of 0.8, at which time the medium was washed away with 10 mM MgSO_4_ and the OD_600_ was adjusted to 0.5 for rhizobial inoculation.

BR sensitivity assays were conducted by growing soybeans to the VC stage. To determine how eBL affects soybean nodule development, a nitrogen-deficient nutrient solution containing eBL was added in place of the initial nutrient solution, changing this medium once per week, whereas control plants instead received an equivalent volume of nitrogen-deficient nutrient solution with ethanol. On day 30 post-inoculation, nodule number and nodule dry weight were assessed.

### 2.3 Infection event analyses

Acetone was used to fix select lateral roots from eBL-treated or control soybean plants on day 24 post-inoculation with HH103. As the β-glucuronidase reporter gene was encoded by the HH103-GUS strain, GUS staining was conducted as in prior reports after using 75% alcohol for decolorization. The utilized GUS staining solution consisted of 1 mM potassium ferricyanide, 1 mg/L X-gluc, and 100 mM sodium phosphate (pH=7.5). These analyses were performed using lateral roots from near the root-stem interface, with infection events being visualized and counted via light microscopy (Leica LM2500, Germany). These analyses were conducted using three biological replicates.

### 2.4 qRT-PCR

Rhizobia-inoculated soybean roots were collected, crushed in liquid nitrogen, and the FreeZol Reagent (Vazyme, China) was used for RNA extraction, followed by the use of a HiScript II 1st Strand cDNA Synthesis Kit (Vazyme) to produce cDNA. cDNA then used for qPCR analyses performed with a Roche 480 instrument (Stratagene, CA, USA) and the ChamQ Universal SYBR qPCR Master Mix (Vazyme, Nanjing, China). The 2^^(-ΔCT)^ algorithm was used to assess relative gene expression, using *GmUNK1* (*Glyma.12g020500*) for normalization (Ma et al., 2024b).

### 2.5 RNA-Seq

Genes responsive to BRs at the time symbiosis is established were identified by isolating total RNA from the roots of eBL-treated DN50 seedlings inoculated with HH103 on day 24 post-inoculation. The TruePrep RNA Library Prep Kit for Illumina (Vazyme) was used for library preparation, followed by RNA sequencing. The resultant data for each sample were assembled with the Cufflinks or StringTie software. Gene and transcript levels were separately quantified with RSEM, and expression levels were normalized with DEseq2 to conduct differential expression analyses according to a negative binomial distribution (Love et al., 2014). Differentially expressed genes (DEGs) were identified as those with a normalized fold change > 2, a *p*-value < 0.01, and a false discovery rate (FDR) < 0.01.

### 2.6 Hairy roots transformation

*A. rhizogenes* strain K599 containing the pSoy10 *GmUbi3:GmWRKY33a-GFP* or B7gWWIWG2(II)-*GmWRKY33a* vectors were used for soybean hairy root transformation, using empty vector (EV1 or EV2)-containing strains as a control. A portable fluorescent protein excitation light source (LUYOR) was used to identify transgenic roots, removing any roots that were not transgene-positive (Ma et al., 2024a). The confirmation of GmWRKY33a silencing or overexpression in these roots was achieved by qPCR. Positive hairy roots were then inoculated with HH103 under conditions of eBL treatment, using an equal volume of ethanol-containing nutrient solution as a control. On day 30 post-inoculation, nodulation testing was performed. Three independent experiments with 25 biological replicates each were used when assessing nodulation phenotypes.

## 3 Results

### 3.1 BRs suppress the formation of infection threads during the establishment of symbiosis

**Figure 1:**
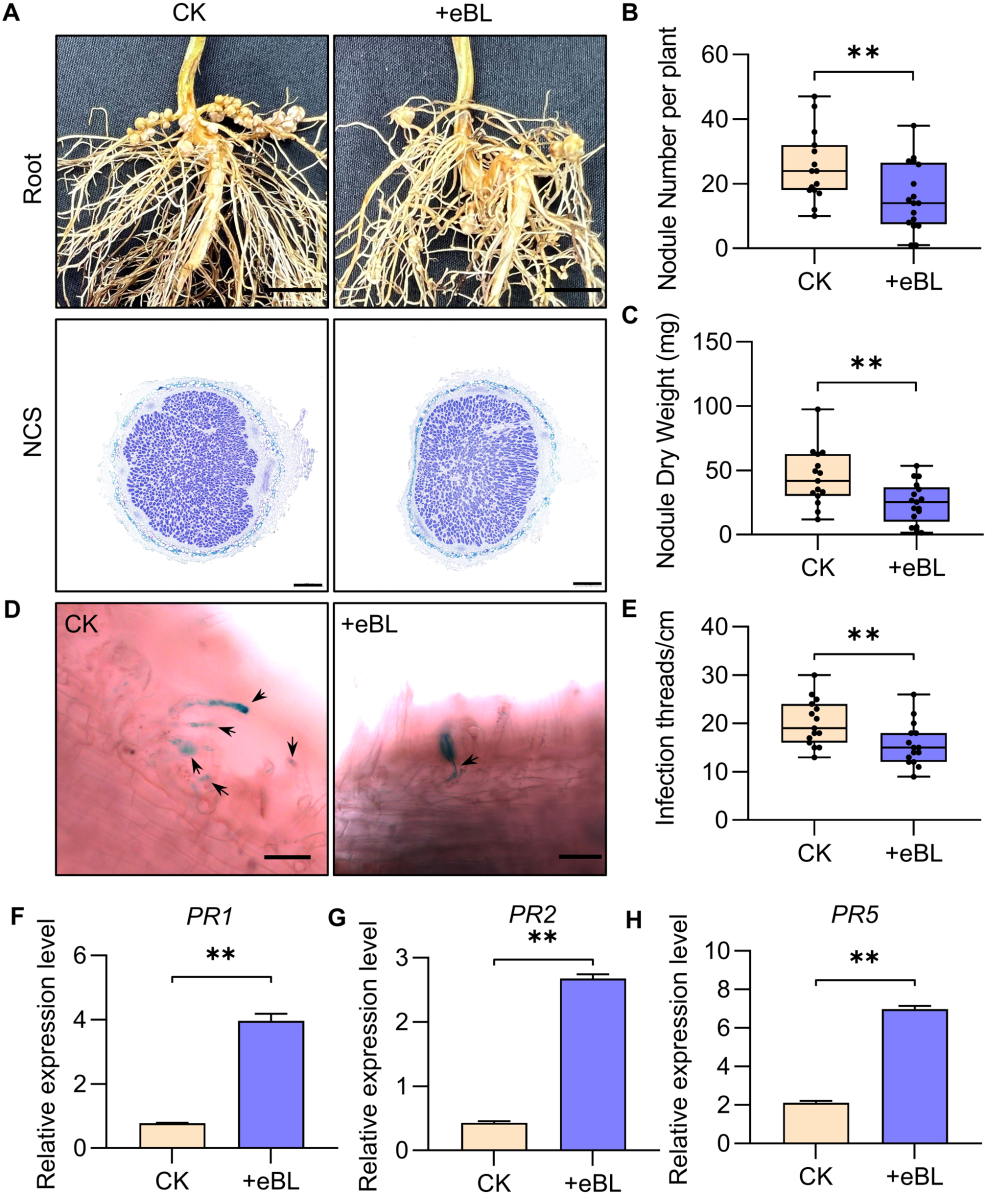
BR suppresses the formation of root nodules in DN50 soybean plants. **(A)** The root and nodular phenotypes of soybean plants subjected to eBL or CK treatment. Scale bars: 1 cm for roots, 500 µm for nodule cross-sections (NCS). **(B, C)** Nodule number **(B)** and nodule dry weight **(C)** values for the plants shown in (A). **P < 0.01, Student’s *t*-test (*n*=15). **(D)** Infection thread (IT) penotypes in DN50 plants inoculated with HH103-GUS on 1 dpi under eBL treatment or CK conditions. Scale bar: 50 µm. **(E)** IT numbers per cm for DN50 plants from (D). **P < 0.01, Student’s t-test (n=15). **(F–H)** Relative defense-related gene (*GmPR1*, *GmPR2*, *GmPR5*) expression under conditions of eBL or CK treatment was assessed with the 2^-ΔCt^ method, with *GmUNK1* (*Glyma.12g020500*) for normalization. Data were compared with Student’s *t*-tests (*n*=3), **P < 0.01.

In the Williams 82 cultivar, BRs have previously been shown to inhibit soybean nodule formation and root development (Hunter, 2001). Consistently, significant reductions in nodule number and nodule dry weight were observed for the root systems of epibrassinolide (eBL)-treated soybean plants as compared to controls (**Figures 1A–C**). Nodule cross-section (NCS) staining with toluidine blue did not reveal any apparent differences in the numbers of cells infected within these established nodules (**Figure 1**). This suggests that while BRs suppress root nodule formation, their effects on the later stages of nodule development are less pronounced. Quantification of the infection threads (ITs) revealed that their numbers were significantly reduced following eBL treatment (**Figures 1D, E**). This supports the ability of BRs to primarily impact nodule formation through their effects on IT formation.

The role that signaling pathways downstream of BRs play in symbiosis establishment was assessed by evaluating relative symbiosis-related (*NIN1a*, *NSP1a*, and *ERN1*) and defense-related (*PR1*, *PR2*, and *PR5*) gene expression 24 h after infection. This approach revealed that treatment with BRs led to the downregulation of symbiosis-related genes together with defense-related gene upregulation (**Figures 1F–H**; **Supplementary Figure S1**). This suggests that the ability of BRs to regulate symbiosis establishment is related to their regulatory effects on both symbiosis-associated genes and defensive responses.

### 3.2 Identification of eBL treatment-related DEGs associated with the establishment of symbiosis

**Figure 2:**
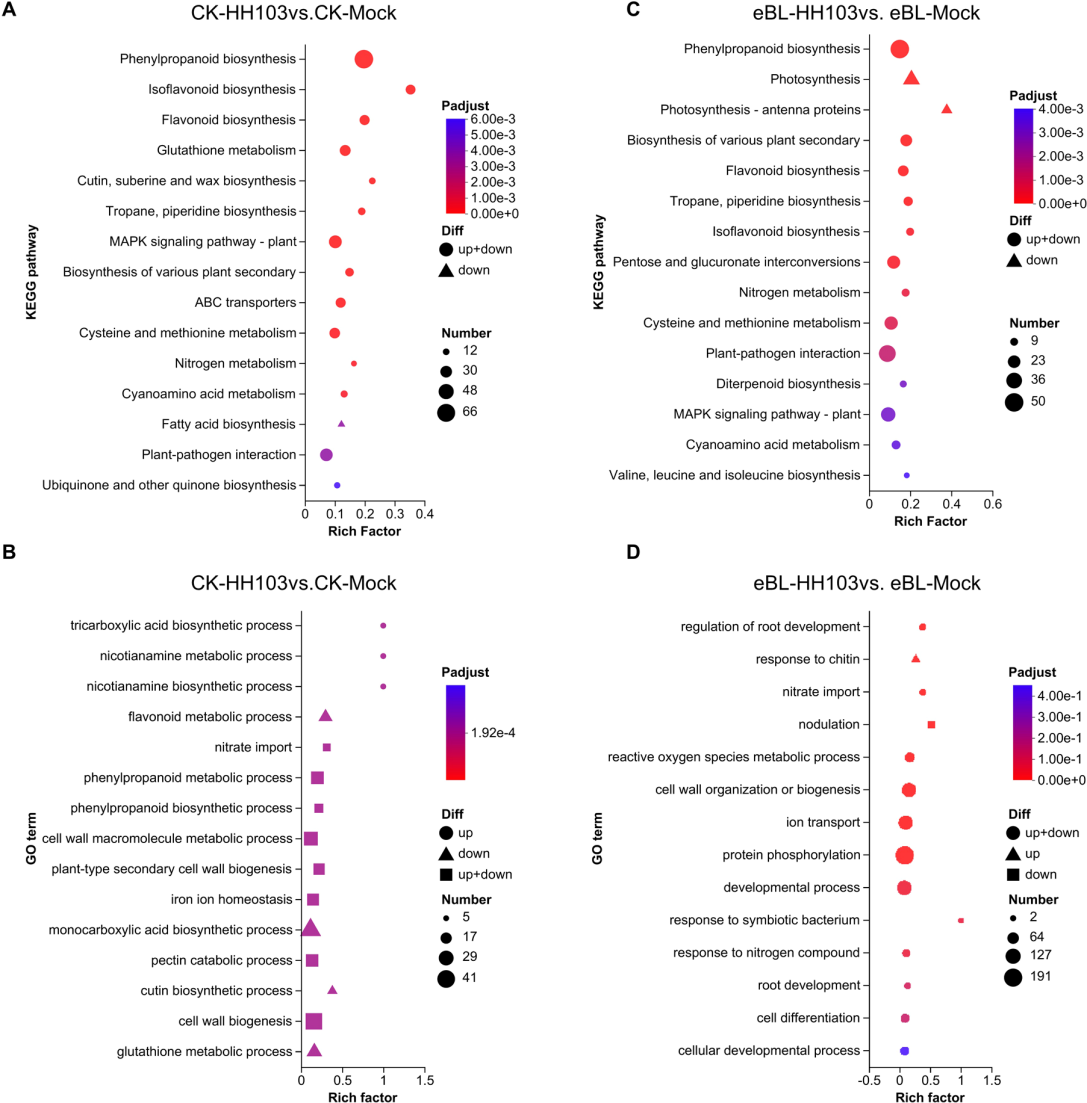
Functional enrichment analyses of DEGs identified under conditions of eBL treatment. **(A, B)** KEGG **(A)** and GO **(B)** enrichment analyses of DEGs identified when comparing the CK-HH103 and CK-Mock groups. **(C, D)** KEGG **(C)** and GO **(D)** enrichment analyses of DEGs identified when comparing the eBL-HH103 with eBL-Mock groups.

To gain additional insight into how BR signaling shapes symbiosis establishment, RNA-seq analyses of roots from DN50 seedlings inoculated with HH103 or MgSO_4_ (mock control) under eBL or control treatment conditions were next conducted. In the absence of eBL, HH103 inoculation led to the upregulation of 727 DEGs and the downregulation of 1,234 DEGs (**Supplementary Figure S2A**, **Supplementary Table S1**). ‘KEGG (Kyoto Encyclopedia of Genes and Genomes)’ enrichment analyses of these DEGs revealed that the downregulated genes were primarily enriched in the fatty acid biosynthesis pathway (**Figure 2A**). ‘GO (Gene Ontology)’ enrichment analyses further indicated that downregulated DEGs were enriched in the flavonoid metabolic process, monocarboxylic acid biosynthetic process, cutin biosynthetic process, and glutathione biosynthetic process terms, together with the significant downregulation of the cutin biosynthetic process and glutathione metabolic process pathways (**Figure 2B**). HH103 inoculation under conditions of eBL treatment was associated with 1,206 and 1,456 upregulated and downregulated DEGs, respectively (**Supplementary Figure S2A**, **Supplementary Table S2**). KEGG enrichment analyses of these genes revealed that they were primarily enriched in the phenylpropanoid biosynthesis and plant-pathogen interaction pathways (**Figure 2C**), while also being enriched in the protein phosphorylation and developmental process GO terms (**Figure 2D**). Chitin response-related genes were significantly upregulated, while nodulation-related genes were significantly downregulated. These DEG expression patterns and enriched pathways may play important roles in the effects of BRs on IT formation.

### 3.3 BRs promote immune-related gene upregulation during symbiosis establishment

**Figure 3:**
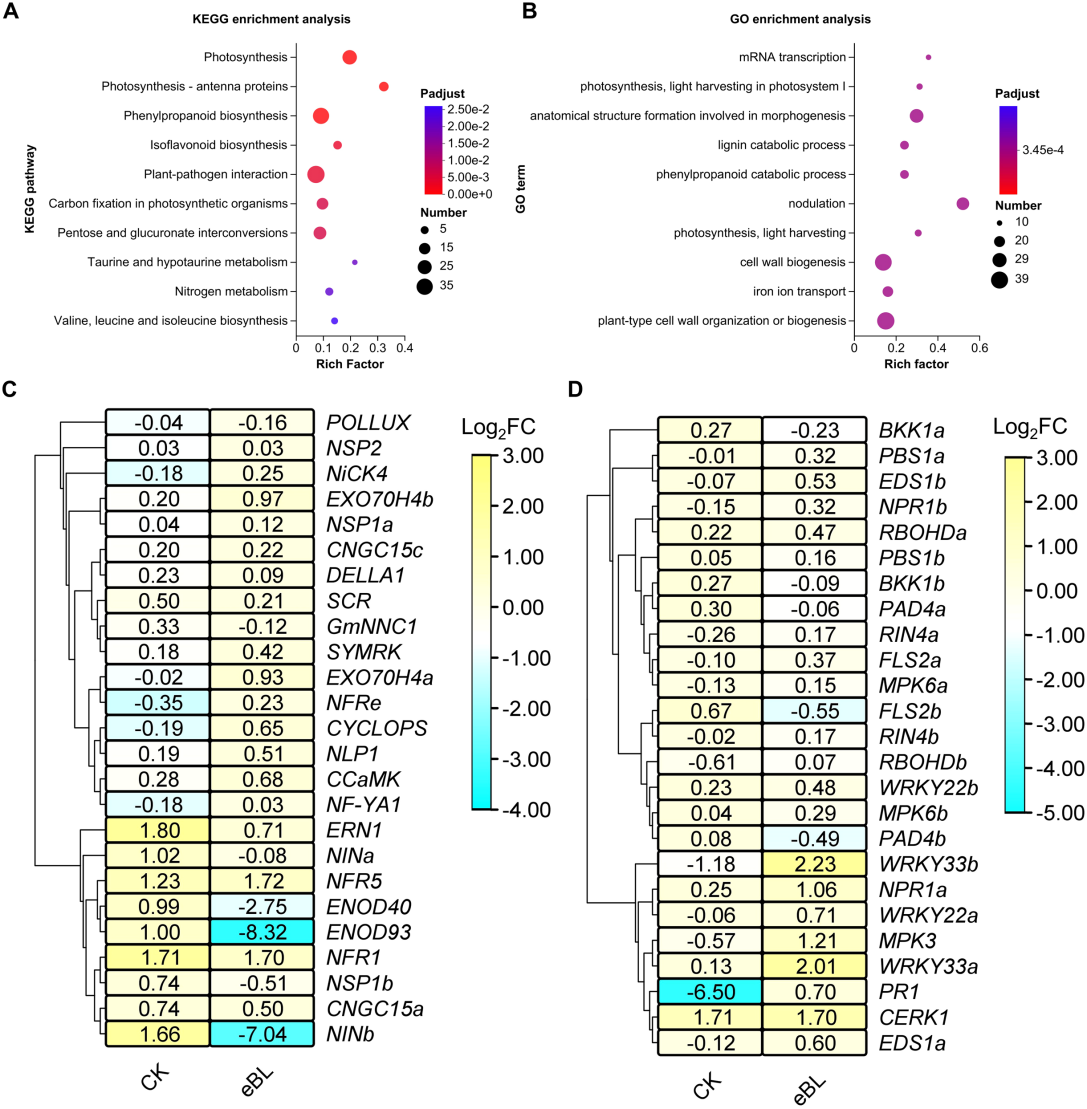
DEGs associated with responses to BR signaling in the context of symbiosis establishment. **(A, B)** KEGG **(A)** and GO **(B)** enrichment analyses of DEGs responsive to BR signaling. **(C)** A hierarchically clustered heat map of genes involved in shaping symbiosis establishment in roots under conditions of eBL treatment. Data are given as Log_2_FC (Fold change) values. **(D)** A hierarchically clustered heat map of genes involved in regulating the immune status of plant roots under conditions of eBL treatment. Data are given as Log_2_FC values.

In order to gain a deeper insight into BR-related signaling mechanisms involved in the initiation of symbiosis and the associated genetic components, RNA-sequencing data were meticulously examined. This analysis revealed 1,074 genes that were upregulated and 1,362 genes that were downregulated in response to BRs during the symbiosis establishment process (**Supplementary Figure S2B**, **Supplementary Tables S3**, **4**). These genes were primarily found to be involved in the plant-pathogen interaction and phenylpropanoid biosynthesis pathways (**Figure 3A**). These BR-responsive DEGs were also involved in plant cell wall organization or biology, morphogenesis-related anatomical structure formation, and nodulation process GO terms (**Figure 3B**).

As the establishment of a symbiotic relationship between soybean plants and rhizobia hinges on symbiotic gene activation and the inhibition of immune-related genes, and most BR-induced DEGs identified above were enriched in immunity and symbiosis-related processes, the effects of BRs on these signaling pathways were further explored by screening for genes associated with both symbiosis establishment and plant immunity under conditions of BR exposure. This approach revealed that BR treatment led to the downregulation of symbiosis-associated genes including *NIN1b*, *ENOD93*, and *ENOD40* (**Figure 3C**), together with the upregulation of immune genes that included *WRKY33s*, *PR1, PBS1s*, and *EDS1s* following HH103 inoculation (**Figure 3D**). These data offer evidence in support of the ability of BRs to inhibit symbiotic nodulation through the suppression of most symbiotic gene expression and by disrupting the ability of soybean plants to hinder immune-related gene expression in the context of symbiosis establishment.

### 3.4 WGCNA-based identification of symbiosis-related gene expression following BR exposure

**Figure 4:**
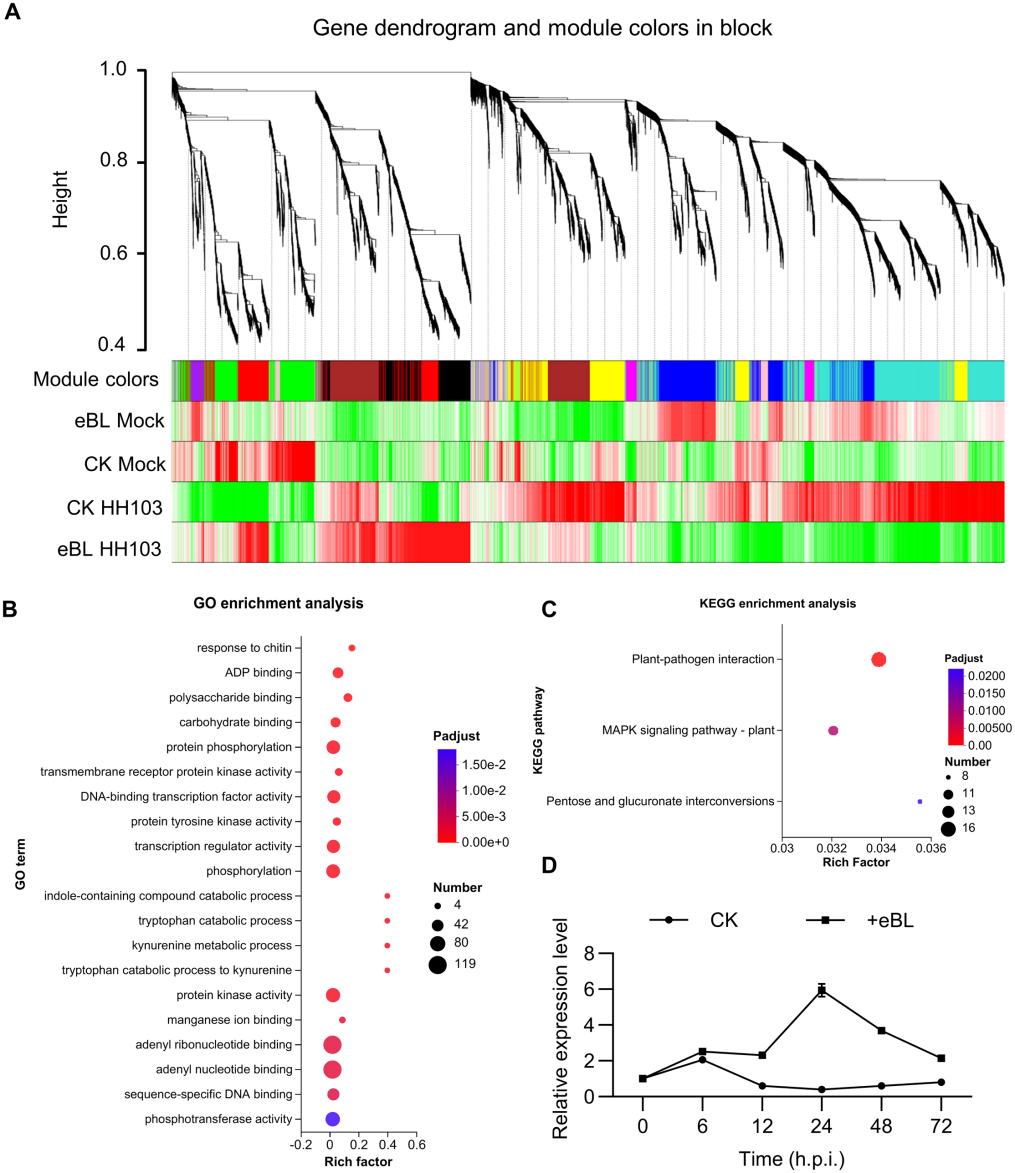
Identification of genes responding to BR after inoculation with HH103 by weighted gene coexpression network analysis. **(A)** A clustering dendrogram for genes showing the original and assigned colors of established modules. Each leaf in the dendrogram represents a gene. **(B, C)** GO **(B)** and KEGG **(C)** enrichment analyses of the genes in the MEblack module. **(D)** Relative *GmWRKY33a* expression under eBL treatment conditions during the establishment of symbiosis was assessed with the 2^-ΔCt^ method, using *GmUNK1* for normalization.

To gain further insight into the genes involved in the responses of soybean plants to BR treatment during the establishment of symbiosis, a weighted gene co-expression network analysis (WGCNA) was next conducted after screening those genes with low expression levels (FPKM < 0.01) from the RNA-seq dataset. This led to the classification of 9,671 genes into 13 co-expression clusters according to their correlations, with different colors being assigned to each module (**Figure 4A**). Trait-specific modules were selected at a *P*-value < 0.05, including four that were associated with HH103 responses. Of these, two modules were respectively enriched for BR signaling and HH103 responses (MEblack and MEred, respectively), with the MEblack and MEred modules respectively containing 780 and 862 genes (**Supplementary Figure S3**, **Supplementary Table S5**). Gene significance analyses of these modules revealed a significant correlation between the MEblack module and HH103 inoculation under BR treatment conditions (**Supplementary Figures S4A, B**), whereas it did not exhibit a significant correlation with HH103 inoculation in the absence of BR treatment. This suggests that the genes contained within the MEblack module may play a significant role in BR signaling responses during the establishment of symbiosis such that they were selected as the focus for further study. GO enrichment analyses of this gene module revealed that these genes were primarily involved in the adenyl ribonucleotide binding, adenyl nucleate binding, and protein kinase activity processes (**Figure 4B**). KEGG enrichment analyses also supported the enrichment of these genes in the plant-pathogen interaction and plant MAPK signaling pathways (**Figure 4C**; **Supplementary Figures S5A, B**).

### 3.5 *GmWRKY33a* is a hub gene responsive to BR signaling

Next, a module membership threshold of eigengene based connectivity (kME > 0.95), determined based on gene connectivity, was used to screen for the top 30 hub genes within the MEblack module, after which they were subjected to functional annotation (**Supplementary Figure S6A**, **Supplementary Table S6**). These analyses revealed that *Glyma.09G280200*, which encodes a GmWRKY33 protein (designated GmWRKY33a) exhibited high levels of connectivity and significant enrichment in the plant-pathogen interaction and plant MAPK signaling pathways (**Supplementary Figure S6B**). Given the importance of immune suppression in the context of symbiosis establishment, the ability of BRs to induce immune responses in leguminous plants during this process, and the status of WRKY33 as a key transcription factor associated with immune-related signaling, it was selected as the target for further study. In subsequent qPCR analyses, HH103 inoculation was found to significantly increase *GmWRKY33a* expression under conditions of eBL treatment relative to untreated plants (**Figure 4D**). These data are support that *GmWRKY33a* serving as a hub gene responsive to BR signaling in the establishment of symbiosis.

### 3.6 BR signaling suppresses nodulation via *GmWRKY33a*-mediated immune signaling activity

**Figure 5:**
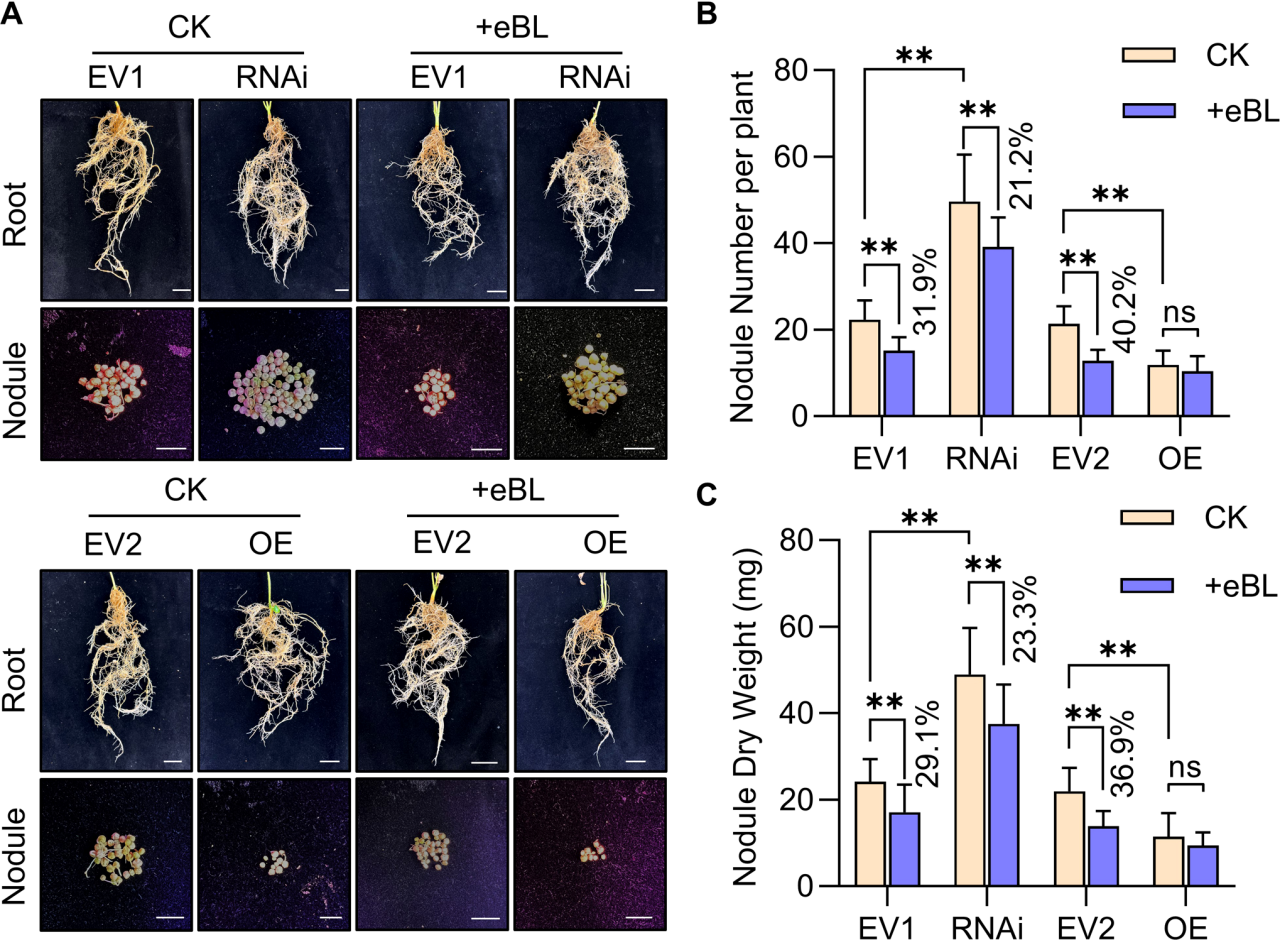
The effect of *GmWRKY33a* knockdown or overexpression on nodule phenotypes. **(A)** Nodule phenotypes were assessed for hairy roots following EV1, RNAi, EV2, or OE construct transformation and eBL or CK treatment. **(B, C)** Nodule number **(B)** and nodule dry weight **(C)** were analyzed for roots established as in **(A)**. Results were compared with Student’s *t*-tests (*n*=25), **P < 0.01; ns, not significant. Percentages denote decreases or increases as compared to CK.

To determine the impact of *GmWRKY33a* on soybean symbiotic nodulation and BR signaling activity, *Agrobacterium rhizogenes* K599 carrying the antisense pB7gWWIWG2(II)-*GmWRKY33a* construct was used to silence this gene. Alternatively, *GmWRKY33a* overexpression (OE) was achieved with a K599 strain harboring the *GmUbi3: GmWRKY33a-GFP* construct. Soybean hairy roots were transformed with these strains or empty vector controls (EV1 or EV2), confirming successful *GmWRKY33a* OE or knockdown in these hairy roots via qPCR (**Supplementary Figure S7**). Nodule phenotype analyses revealed that *GmWRKY33a* silencing led to significant increases in root nodule numbers and dry weight, whereas its OE reduced the number of nodules (**Figure 5**), consistent with a role for GmWRKY33a as a negative regulator of the establishment of symbiosis. Following treatment with eBL, *GmWRKY33a* silencing led to less numbers and dry weight as compared to EV1 transformation, but the decrease in the nodule number and dry weight of these hairy roots relative to that for EV1, and there was no significant difference in nodule numbers for hairy roots overexpressing *GmWRKY33a* following eBL treatment relative to the corresponding control (**Figure 5**).

**Figure 6:**
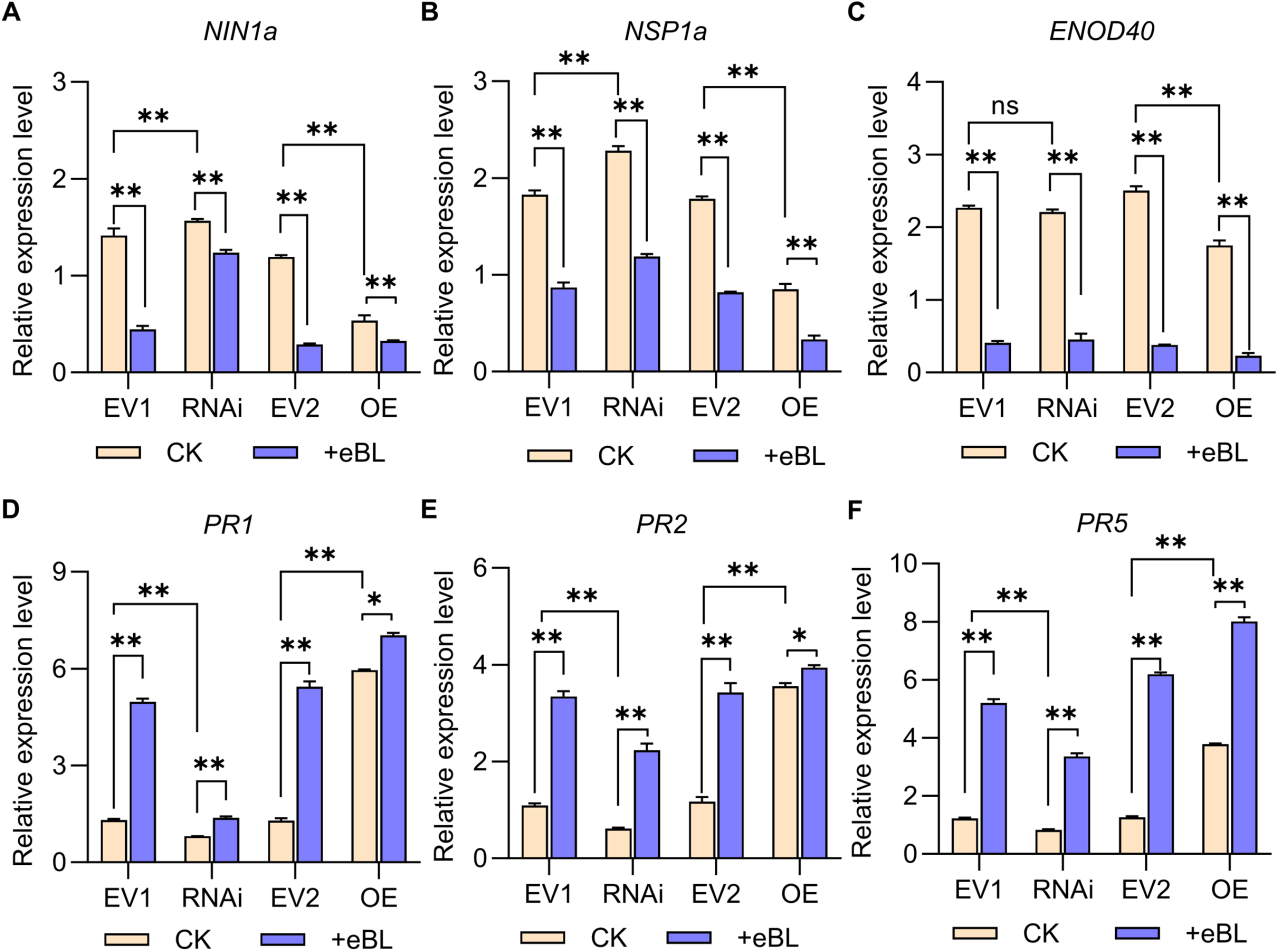
Relative symbiosis and immune-related gene expression. **(A–F)** the 2^-ΔCt^ method was employed to assess relative expression (*NINa*, *NSP1a*, *ENOD40*, *PR1*, *PR2* and *PR5*), with *GmUNK1* (*Glyma.12g020500*) for normalization. Data were compared with Student’s *t*-tests (*n*=3), *P < 0.05; **P < 0.01; ns, not significant.

Relative symbiosis and immune-related gene expression was also analyzed at 24 h post-infection in transgenic hairy roots, revealing that silencing *GmWRKY33a* promoted *NIN1a* and *NSP1a* upregulation without affecting *ENOD40* relative to EV1, while overexpressing *GmWRKY33a* led to the downregulation of *NIN1a*, *NSP1a*, and *ENOD40* (**Figures 6A–C**). Transgenic hairy roots in which *GmWRKY33a* was silenced exhibited *NIN1a*, *NSP1a*, and *ENOD40* downregulation following eBL treatment, with the downregulation of *NIN1a* and *NSP1a* being less than that for EV1 hairy roots under eBL treatment conditions (**Figures 6A–C**). The immune-related *PR1*, *PR2*, and *PR5* genes exhibited expression patterns opposite those of *NIN1a* and *NSP1a*, with *GmWRKY33a* silencing reducing their expression (**Figures 6D–F**). Treatment with eBL in soybean hairy roots in which *GmWRKY33a* was overexpressed led to increases in the expression of these target genes relative to CK (Control Check) treatment, while this increase was smaller relative to EV2 transfection (**Figures 6D–F**). Given the importance of WRKY33a as an immune-related transcription factor, these data support the ability of BR signaling to inhibit the establishment of symbiosis through the upregulation of *GmWRKY33a* and the modulation of *NIN1a*, *NSP1a*, and immune-related gene expression.

## 4 Discussion

BRs are a key class of phytohormones responsible for controlling the growth and development of plants (Santner and Estelle, 2009). The present data highlight the ability of BRs to suppress symbiotic nodule formation, although the effects of BRs on nodulation have been reported to vary across leguminous species such that they are positive and negative in particular contexts. These effects may be attributable to differences in BR concentration, BR composition, differences in growth states, or rhizobia genotypes, and the specific genotypes that regulate these effects are poorly understood. Previous studies have demonstrated that BR signaling via GmBES1 suppresses GmNSP1 activity and adjusts NF signaling to affect symbiosis (Chen et al., 2023), while this study suggests that BR signaling can activate immune-related gene expression to suppress the establishment of symbiosis. As establishing symbiosis necessitates activating plant symbiotic signaling while inhibiting certain immune-related genes, this may partially account for the dual roles that BRs exert in pea plants during different stages of nodulation. The GmBS1-1 homolog GmBEHL1 is also capable of interacting with GmNNC1, thereby inhibiting the establishment of symbiosis and affecting nodule numbers and IT numbers (Yan et al., 2018). These data indicate that BRs may thus regulate nodulation in legumes through a range of signaling processes and associated mechanisms.

Here, preliminary analyses revealed that BRs were capable of promoting *GmPR1*, *GmPR2*, and *GmPR5* upregulation. Additional transcriptomic analyses indicated that these BRs were capable of inducing the expression of many immune-related genes in DN50 plants in the context of symbiosis establishment. A growing body of evidence suggests that the failure to repress the expression of these genes can compromise the establishment of symbiosis between soybean plants and rhizobia. BAK1 is an important regulator of pathogenesis in plants, serving as a co-receptor for many microbial pattern recognition receptors that coordinates pathogen responses and serves as a key BR signaling-related receptor (Halter et al., 2014; Shang et al., 2016; Imkampe et al., 2017). Once symbiosis has been established, SymRK interacts with and inhibits BAK1 to strike a balance between symbiosis and pathogenesis (Feng et al., 2021). The upregulation of the *GmMPK3* and *GmMPK6a/b* kinases downstream of BAK1 is indicative of BAK1-mediated immune response activation. The dysregulation of BAK1 in the roots of soybean plants during the establishment of symbiosis may account for the lower numbers of nodules and ITs observed in this study. BR treatment can induce the expression of the salicylic acid (SA) signaling pathway marker *GmPR1* during symbiosis establishment, and BR signaling in *Arabidopsis* can coordinate SA responses and shape host immunity (Liu et al., 2022). BR-induced SA receptor *GmNPR1* expression during symbiosis establishment also suggests the ability of BR signaling to shape SA signaling and to synergistically modulate interactions between soybean roots and rhizobia. This BR signaling can influence SA signaling-related immune functionality in these plants, highlighting an important topic for further study. PBS1 can serve as another important receptor related to plant immune activity (Swiderski and Innes, 2001), interacting with NopT, a rhizobial type III effector, to control soybean immunity during rhizobial infection. *GmPBS1* is another hub gene that impacts the responses of soybean plants to the rhizobial type III effectors NopT and NopP (Khan et al., 2022; Li et al., 2023), with its significant upregulation in response to BR treatment supporting the ability of these phytohormones to modify the susceptibility of legume plants to pathogen infection, altering downstream immune signaling activity and thus explaining the ability of BR signaling to inhibit nodulation. Whether rhizobial type III effectors, which are important signals for the establishment of symbiosis, and involved in or impact BR signaling and the degree to which they are involved in immune activation mediated by BR during this process will need to be studied at length in the future. Notably, BR treatment suppressed many key symbiosis-related genes including *NIN1s*, *ERN1*, *ENOD40*, and *ENOD93*, although it did upregulate some symbiotic receptors, such as *SymRK* and *NFR5*, suggesting a need for additional mechanistic research aimed at better understanding these findings.

Through transcriptomic WGCNA analyses, genes included in the MEblack module were found to be primarily associated with responses to BR signaling and the control of soybean immune signaling against rhizobium during symbiosis establishment. Of the targets within this module, GmWRKY33a was previously identified as an important regulator of pathogen resistance that serves as a substrate for CDPK5/6 and MPK3/6 (Zhou et al., 2020). While some reports have found *GmWRKY33* to be upregulated during symbiosis establishment, the mechanisms through which it impacts symbiotic signaling have not been clarified. Here, GmWRKY33 silencing was found to attenuate the impact of BR on nodule numbers, suggesting that knocking down *GmWRKY33a* may disrupt BR-driven immune signaling during symbiosis establishment, thereby limiting the impact of this phytohormone on symbiotic nodulation. GmWRKY33a is a transcription factor that controls *PR* gene expression (Liu et al., 2018), and treatment with BR induced *GmWRKY33a* expression, at least partially explaining the extensive *PR-*related gene upregulation observed in the RNA-seq dataset. In contrast, *GmWRKY33a* overexpression was associated with the significant downregulation of *NIN1a*, *NSP1a*, and *ENOD40a* in soybean hairy roots relative to EV2 control roots, potentially owing to the ability of GmWRKY33a to promote the upregulation of many immune-related genes and the accumulation of immune-related metabolites, ultimately leading to the loss of homeostatic balance between immunological and commensal signaling regulation in this context. *GmWRKY33a* thus appears to serve as a central hub mediator of BR signaling when symbiosis is being established. *GmWRKY33a* is also a SNAP1/2, SNAP1/2/4, and SNAP1/2/3/4 target (Wang et al., 2023b), although its potential involvement in SNAP-mediated root nodule responses to nitrogen and how BR signaling affects this involvement will need to be studied at length in the future.

In addition to *GmWRKY33a*, the MEblack hub genes *Glyma.01G224800* and *Glyma.05G215900* encode two additional WRKY transcription factors. As WRKY family transcription factors are essential for the regulation of the ability of plants to respond to microbial infections. The patterns of *Glyma.01G224800* and *Glyma.05G215900* expression in response to BR signaling may be similar to those of *GmWRKY33a*. Moreover, *Glyma.02G023800*, *Glyma.05G082500*, and *Glyma.16G136200* were identified as soybean hub genes that were responsive to BR signaling in the setting of symbiosis establishment, encoding leucine-rich repeat-containing disease resistance proteins (CC-NBS-LRR-like). R proteins are directly involved in plant immune responses, and the upregulation of certain R proteins following BR treatment may thus represent one additional process through which BR influences symbiosis. These include the downstream target of GmBZL3, *Glyma.05G082500*, which encodes a BZR1-like protein that serves as a key BR signaling regulator potentially involved in BR responses through the recognition of rhizobial PAMPs (Song et al., 2019), thus leading to the activation of various immune responses following the establishment of symbiosis.

## 5 Conclusion

**Figure 7:**
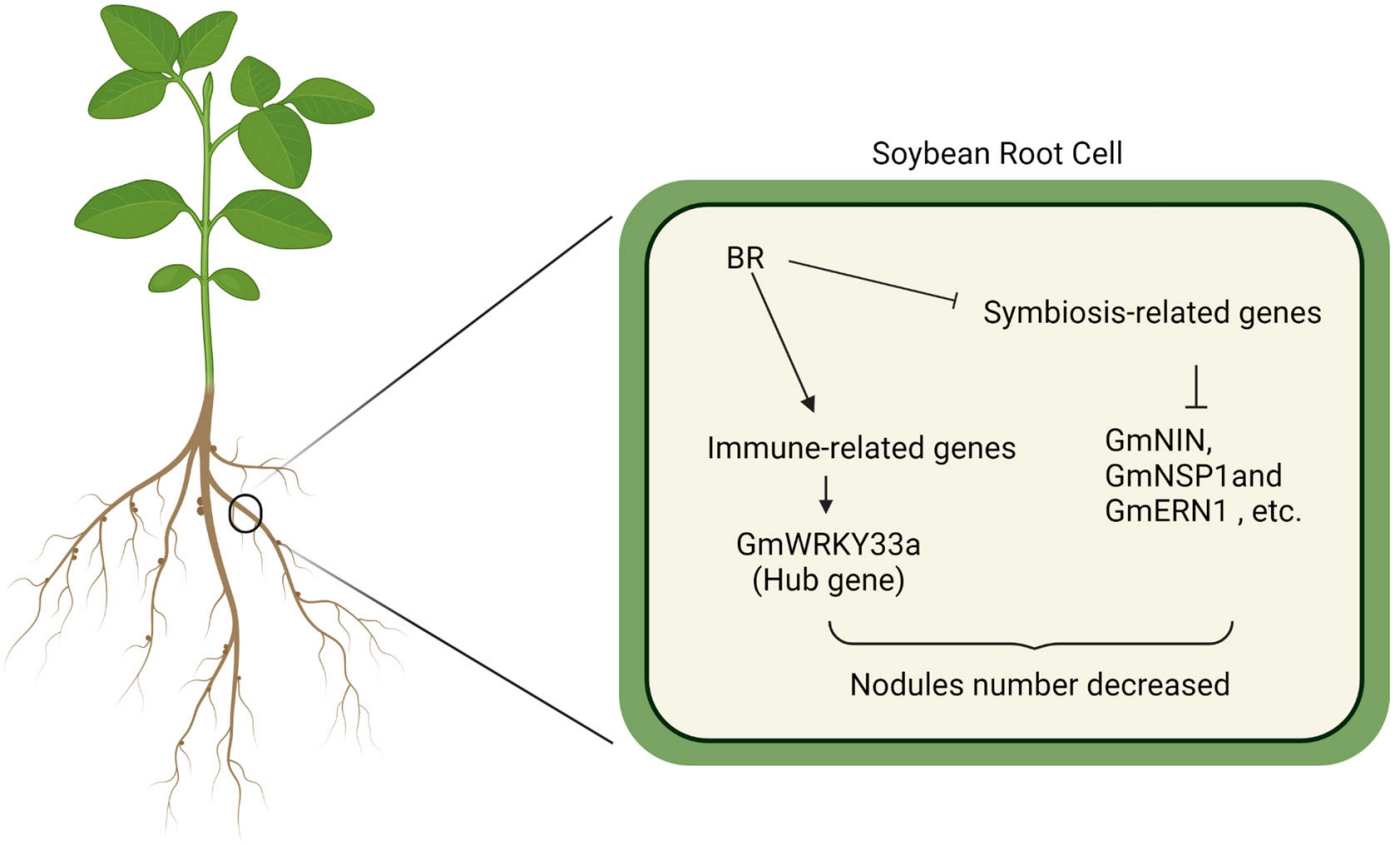
GmWRKY33 acts as a key BR signaling-related transcription factor, negatively regulating the establishment of symbiosis.

The present analysis indicates that BR treatment is sufficient to inhibit the establishment of symbiosis, leading to reduced numbers of nodules and infection threads (IT) in soybean roots. BR signaling induces host immunity, with many differentially expressed genes (DEGs) enriched in immunity-related pathways. Further WGCNA identified *Glyma.09G280200*, which encodes the GmWRKY33 protein, as a central gene involved in BR signaling responses during symbiosis establishment. GmWRKY33 acts as a key BR signaling-related transcription factor, negatively regulating the establishment of symbiosis (**Figure 7**). In summary, these data highlight a novel mechanism through which BR signaling activity governs symbiosis, providing a foundation for efforts to select soybean varieties with more efficient nitrogen fixation.

## Data Availability

All raw sequencing data have been deposited at the NCBI Sequence Read Archive Archive https://www.ncbi.nlm.nih.gov/sra, PRJNA1124407.

## Supplementary material

Supplementary Figure 1 Relative NIN1a, NSP1a, and ERN1 expression. The 2^-ΔCt^ method was employed to assess relative expression, with *GmUNK1* (*Glyma.12g020500*) for normalization. Data were compared with Student’s *t*-tests (*n*=3), **P* < 0.05; ***P* < 0.01; ns, not significant.

Supplementary Figure 2 Identification of DEGs in DB50 roots under conditions of eBL treatment or control conditions. **(A)** Numbers of DEGs at 1dpi with HH103-GUS under eBL treatment or CK conditions. **(B)** Venn diagrams highlighting the numbers of DEGs detected in DN50 samples under conditions of eBL or CK treatment.

Supplementary Figure 3 Module-trait linkage WGCNA analyses. Rows correspond to module-trait genes. Columns correspond to treatments (HH103 inoculation status, eBL treatment status). Cells contain the correlation coefficient and P-values, with color coding as indicated.

Supplementary Figure 5 KEGG pathway maps for MEblack module genes. Genes from the MEblack module exhibited enrichment in the plant-pathogen interaction **(A)** and plant MAPK signaling **(B)** pathways.

Supplementary Figure 6 MEblack module hub genes. **(A)** 30 hub genes from the MEblack module. **(B)** Phylogenetic tree analysis of GmWRKY33a.

Supplementary Figure 7 Relative gene expression in transgenic hairy roots following *GmWRKY33a* silencing or overexpression. ***P*<0.01; ns, not significant; Student’s *t*-test. Error bars indicate the standard deviation.
