# Supplementary figures and images for: GmWRKY33a is a hub gene responsive to brassinosteroid signaling that suppresses nodulation in soybean (Glycine max)
Source: Front Plant Sci. 2025 Jan 16;15:1507307. doi: 10.3389/fpls.2024.1507307 (PMC11779726; doi:10.3389/fpls.2024.1507307)

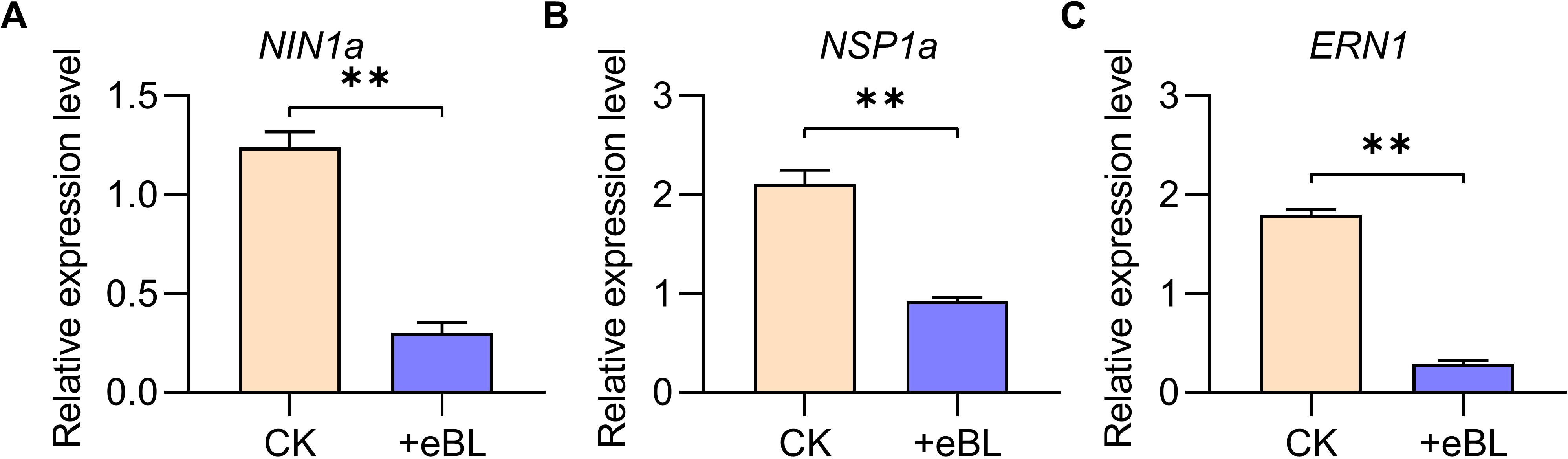

Supplement: Supplementary Figure 1 — Relative NIN1a, NSP1a, and ERN1 expression. The 2-ΔCt method was employed to assess relative expression, with GmUNK1 (Glyma.12g020500) for normalization. Data were compared with Student’s t-tests (n=3), *P < 0.05; **P < 0.01; ns, not significant. [file Image1.jpeg]

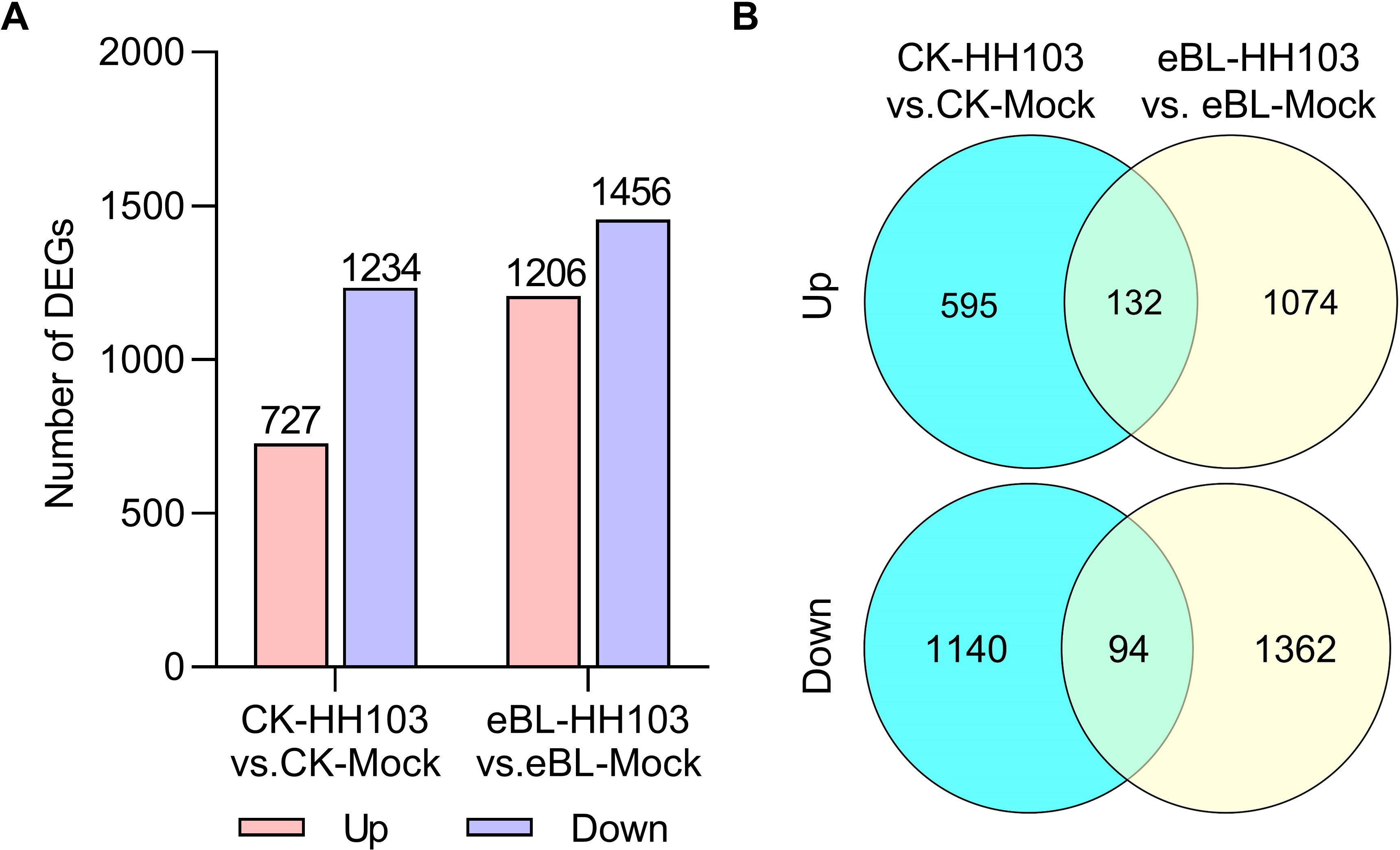

Supplement: Supplementary Figure 2 — Identification of DEGs in DB50 roots under conditions of eBL treatment or control conditions. (A) Numbers of DEGs at 1dpi with HH103-GUS under eBL treatment or CK conditions. (B) Venn diagrams highlighting the numbers of DEGs detected in DN50 samples under conditions of eBL or CK treatment. [file Image2.jpeg]

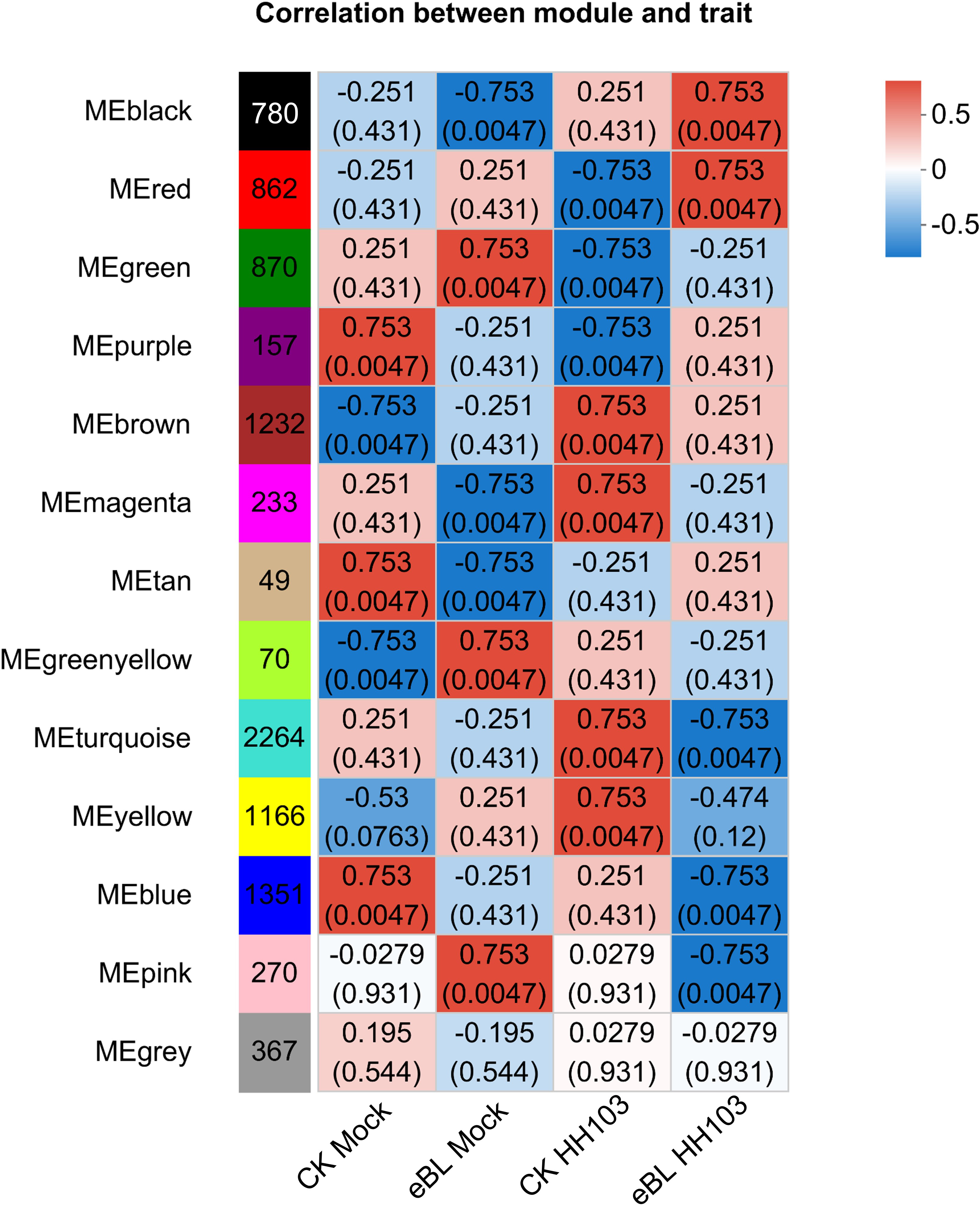

Supplement: Supplementary Figure 3 — Module-trait linkage WGCNA analyses. Rows correspond to module-trait genes. Columns correspond to treatments (HH103 inoculation status, eBL treatment status). Cells contain the correlation coefficient and P-values, with color coding as indicated. [file Image3.jpeg]

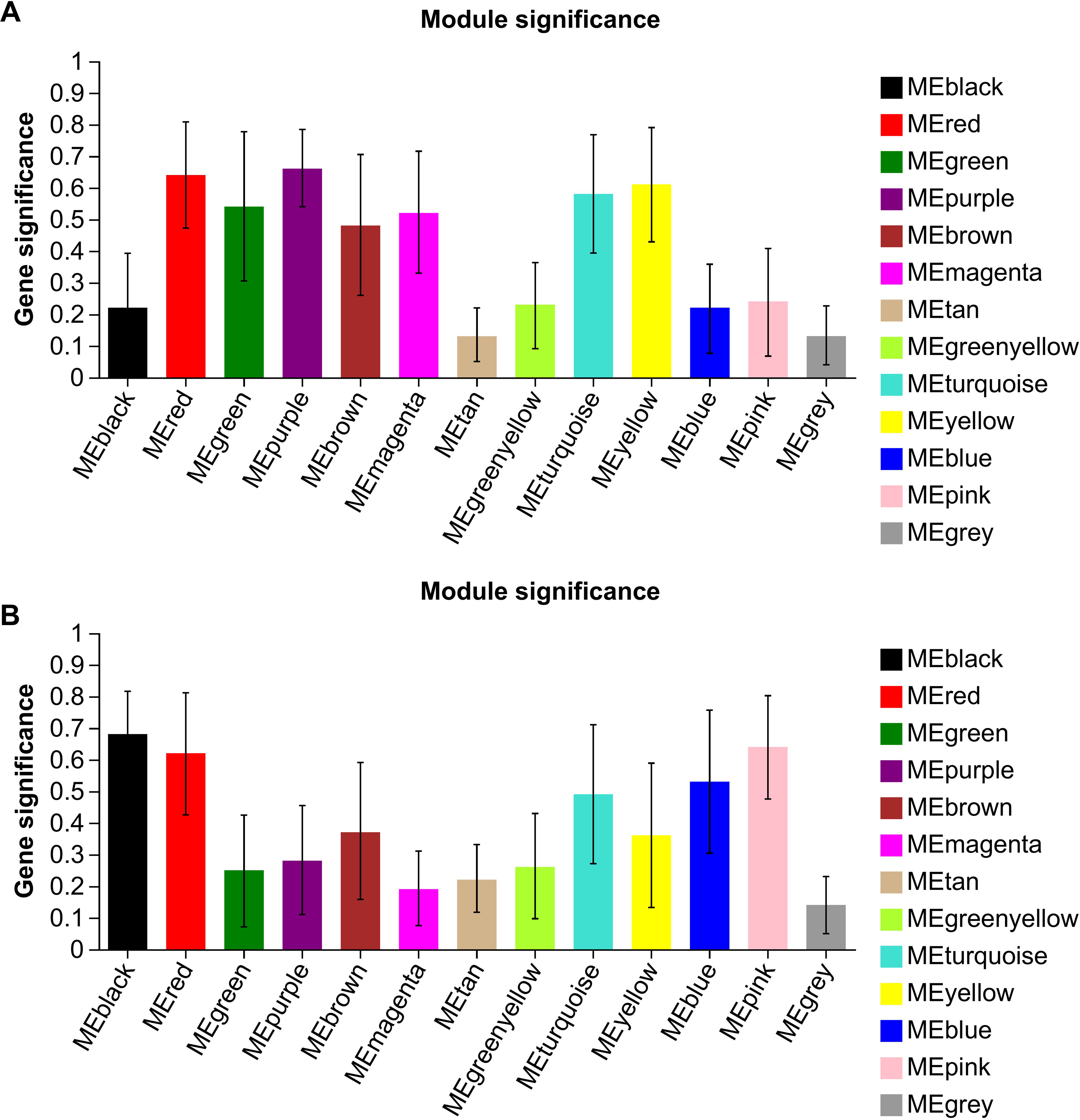

Supplement: Supplementary Figure 5 — KEGG pathway maps for MEblack module genes. Genes from the MEblack module exhibited enrichment in the plant-pathogen interaction (A) and plant MAPK signaling (B) pathways. [file Image4.jpeg]

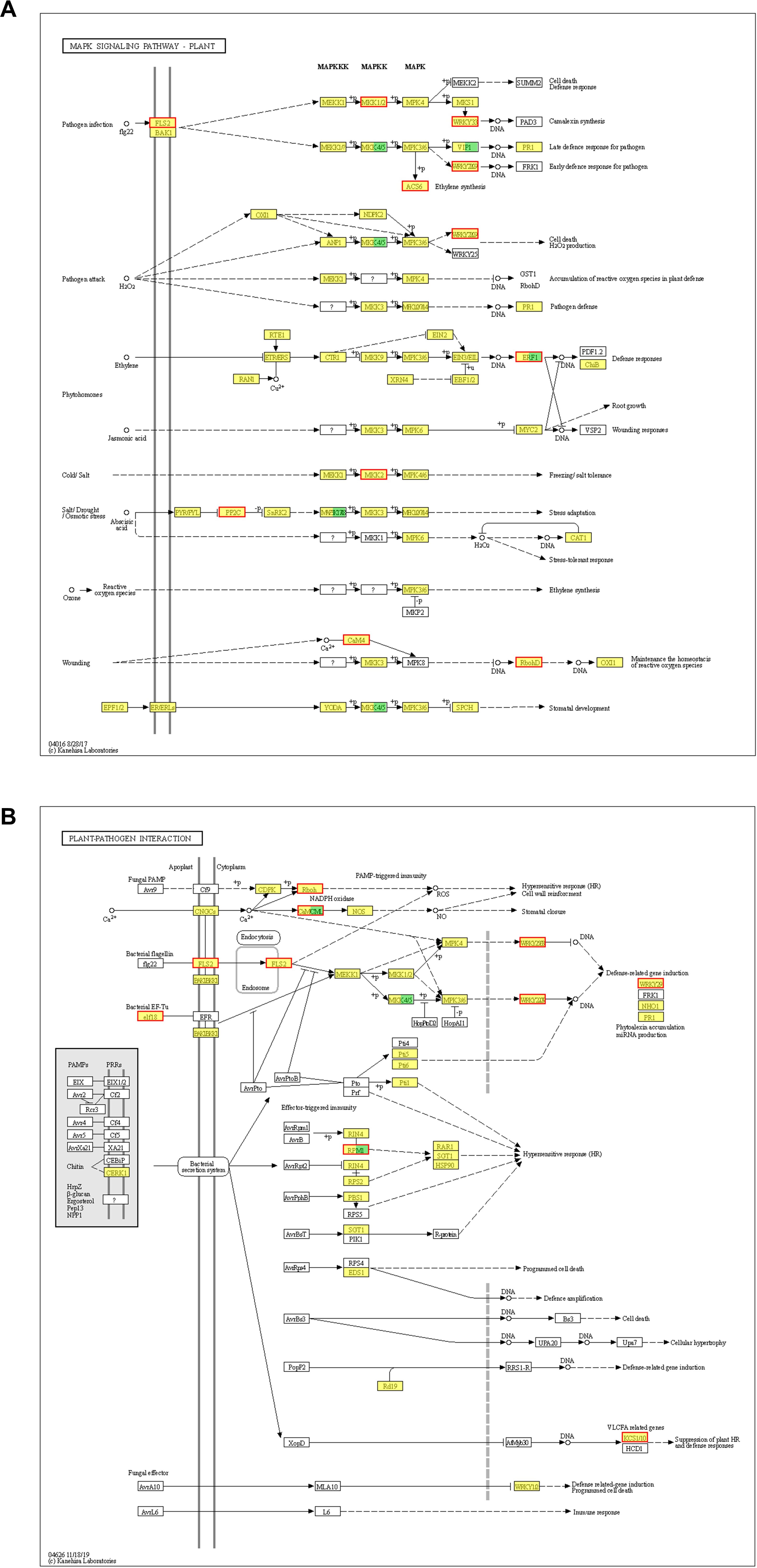

Supplement: Supplementary Figure 6 — MEblack module hub genes. (A) 30 hub genes from the MEblack module. (B) Phylogenetic tree analysis of GmWRKY33a. [file Image5.jpeg]

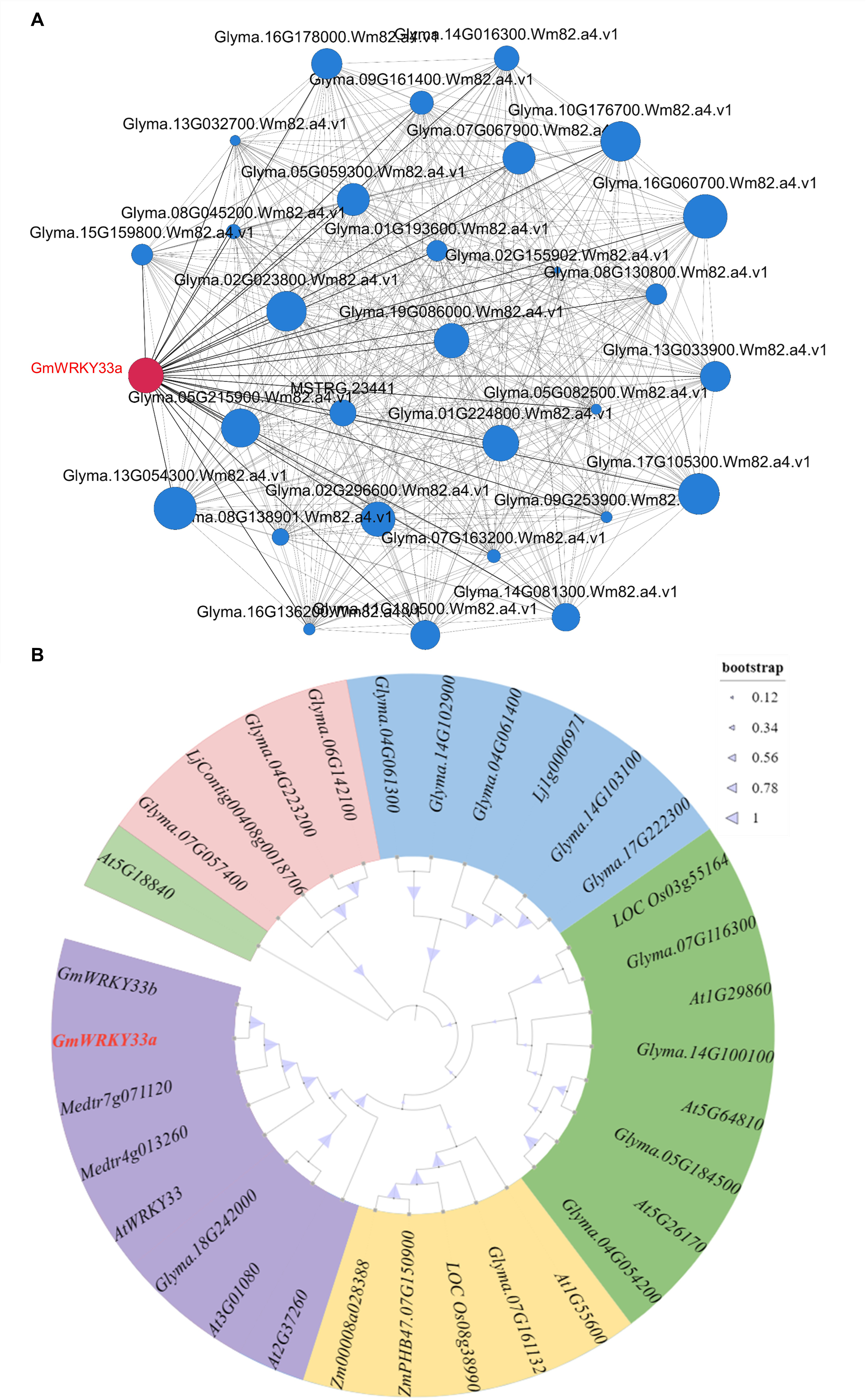

Supplement: Supplementary Figure 7 — Relative gene expression in transgenic hairy roots following GmWRKY33a silencing or overexpression. **P<0.01; ns, not significant; Student’s t-test. Error bars indicate the standard deviation. [file Image6.jpeg]

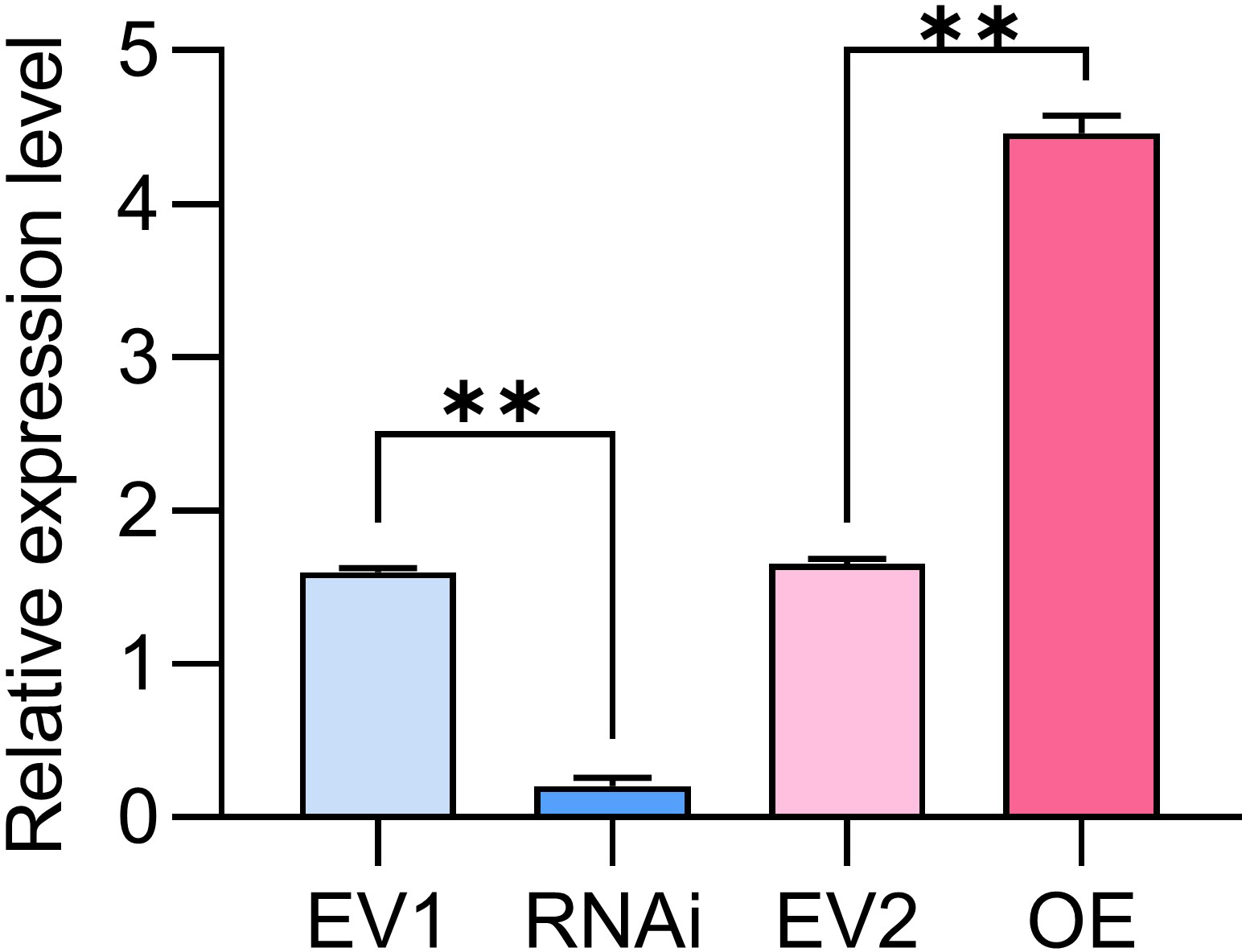

Supplement: Supplementary file 7 [file Image7.jpeg]
